# Coding of obesity in administrative hospital discharge abstract data: accuracy and impact for future research studies

**DOI:** 10.1186/1472-6963-14-70

**Published:** 2014-02-13

**Authors:** Billie-Jean Martin, Guanmin Chen, Michelle Graham, Hude Quan

**Affiliations:** 1 Department of Cardiac Sciences, Libin Cardiovascular Institute, University of Calgary, Room C849, 8th Floor Cardiology, 1403 29th Street NW, Calgary, AB T2N 2 T9, Canada; 2 Department of Medicine, University of Alberta, Edmonton, AB Canada

**Keywords:** Obesity, Coding, Administrative data, Clinical databases, ICD-10

## Abstract

**Background:**

Obesity is a pervasive problem and a popular subject of academic assessment. The ability to take advantage of existing data, such as administrative databases, to study obesity is appealing. The objective of our study was to assess the validity of obesity coding in an administrative database and compare the association between obesity and outcomes in an administrative database versus registry.

**Methods:**

This study was conducted using a coronary catheterization registry and an administrative database (Discharge Abstract Database (DAD)). A Body Mass Index (BMI) ≥30 kg/m^2^ within the registry defined obesity. In the DAD obesity was defined by diagnosis codes E65 – E68 (ICD-10). The sensitivity, specificity, negative predictive value (NPV) and positive predictive value (PPV) of an obesity diagnosis in the DAD was determined using obesity diagnosis in the registry as the referent. The association between obesity and outcomes was assessed.

**Results:**

The study population of 17380 subjects was largely male (68.8%) with a mean BMI of 27.0 kg/m^2^. Obesity prevalence was lower in the DAD than registry **(2.4%** vs. 20.3%). A diagnosis of obesity in the DAD had a sensitivity 7.75%, specificity 98.98%, NPV 80.84% and PPV 65.94%. Obesity was associated with decreased risk of death or re-hospitalization, though non-significantly within the DAD. Obesity was significantly associated with an increased risk of cardiac procedure in both databases.

**Conclusions:**

Overall, obesity was poorly coded in the DAD. However, when coded, it was coded accurately. Administrative databases are not an optimal datasource for obesity prevalence and incidence surveillance but could be used to define obese cohorts for follow-up.

## Background

Obesity is a highly prevalent health concern. While it is well established that many North Americans are obese, [1] similar trends are now being seen worldwide, even in countries such as India, where malnutrition was long the most common nutritional disorder. Obesity is now in line to overtake smoking as the leading preventable cause of morbidity and mortality, causing in excess of 300,000 deaths per year in the United States alone [2,3]. The burden of disease attributable to obesity is in large part due to its impact on the cardiovascular system of these individuals [4-6].

There are several published ways of measuring obesity, ranging from the simple, such as body mass index (BMI, kg/m^2^) or waist circumference, to the complex, including body densitometry and more advanced volumetric techniques such as computed tomography (CT) imaging and magnetic resonance imaging (MRI) [7,8]. While the latter methodologies offer more accurate measurements of body composition, the former are more widely employed due to their relatively low cost, ease of use and familiarity. They are, however, prone to bias: frequently measures of weight and height are taken based on self -report which is rather unreliable, as women tend to underreport weight and men to over report height.

Gathering information on the adiposity of a population is difficult and time consuming: national surveys such as NHANES are expensive, and international studies such as the International Day for the Evaluation of Abdominal Obesity (IDEA) are logistically challenging [9]. It is even more challenging for follow-up studies to collect longitudinal information on obesity and outcomes from a large population. Being able to take advantage of existing administrative data, such as physicians claim and hospital discharge databases, could be potentially time and cost saving because obesity is captured as a diagnosis by the International Classification of Disease (ICD), codes 278 (ICD-9-CM) and E65 – E68 (ICD-10). In many developed countries (such as Canada), there are massive national administrative databases that are easily linked with other databases for research purposes. However, even though BMI is easily derived from standard clinical information, administrative data frequently does not capture height and weight.

There has only been limited evaluation of how frequently obesity is actually captured in administrative databases, or how accurately it is captured. A study by Quan et al from 2003 assessed obesity coding as one of their outcomes [10]. Chart review demonstrated an 8.3% frequency of obesity – while ICD-9-CM data reflected a 2.7% (sensitivity 24.6% and positive predictive value (PPV) 75.9%) rate of obesity, and ICD-10 coding a 1.9% rate (sensitivity 18.6%, PPV83.8%). The association between coded obesity and adverse outcomes has not been well studied in administrative databases, such as those used in Canadian health care systems.

The objective of our study was to assess the validity of obesity coding in an administrative database. To conduct this study, we linked clinically captured physical measurement data, including height and weight, with administrative data to asses how frequently and accurately obesity is captured in an administrative database. To understand performance of obesity research using administrative data, we then determined the association between obesity and outcomes in an administrative database first and then replicate such analysis in registry. We evaluated if results generated from these two databases are comparable. This study will enrich the available information on obesity coding, and will allow the assessment of the utility of administrative data for population surveillance of obesity.

## Methods

### Defining obesity in physical measurement dataset

Our study was conducted using two data sources: The Alberta Provincial Project for Outcomes Assessment in Coronary Heart Disease (APPROACH) database and the Inpatient Discharge Abstract Database for the Calgary health region.

APPROACH is a clinical registry which has captured detailed clinical information on all patients undergoing cardiac catheterization in Alberta, Canada since 1995 [11]. At the time of catheterization, data are collected on clinical risk factors including age, sex, weight, height, body mass index (BMI, kg/m^2^), hypertension, hyperlipidemia, diabetes, chronic lung disease, cerebrovascular disease, congestive heart failure, peripheral vascular disease, renal disease, liver or gastrointestinal disease, and malignancy. Also recorded are the results of coronary catheterization including coronary anatomy and left ventricular ejection fraction, procedures done at the time of initial catheterization and events thereafter (percutaneous coronary intervention (PCI), coronary artery bypass grafting (CABG) and death).

Obesity was defined within the APPROACH database using the Quetelet formula for BMI: weight (kilograms) divided by height (m) squared. A subject was determined to be obese in the APPROACH database if they had a BMI ≥ 30 kg/m^2^. Obesity classes were determined using the standard World Health Organization definitions: subjects with a BMI 30-34.99 kg/m^2^ were considered Obesity Class I, subjects with a BMI 35-39.99 kg/m^2^ were considered Obesity Class II, and subjects with a BMI ≥ 40 kg/m^2^ were considered Obesity Class III [12].

### Defining obesity in the hospital discharge abstract database

The Inpatient Discharge Abstract Database (DAD) collects administrative information on date and time of admission, length of stay and up to 25 diagnoses. Using the DAD for the years 2002-2008, obesity was defined by searching the diagnosis codes E65 – E68 (ICD-10) in the 25 diagnosis coding fields.

The APPROACH database and DAD were linked using Personal Health Numbers (PHNs), which are unique to each individual. Patients were excluded if they were under 18 years of age, did not have a valid Alberta PHN, or were from outside the Calgary Health Region. As the clinical covariates used for the study were obtained from the APPROACH database, cohort entry date was defined as the date of coronary catheterization. Only subjects who had a hospitalization in the first 6 months following catheterization were considered in this study. The diagnosis of obesity in the DAD was ascertained at the time of first hospitalization following catheterization.

### Outcomes variables

The outcomes of interest were all cause mortality, as captured by vital statistics, first hospitalization (any cause) and first cardiac procedure (percutaneous coronary intervention (PCI) and coronary artery bypass grafting (CABG)) in the first year after the date of coronary catheterization. For those patients with multiple admissions in the following year after coronary catheterization, only the first admission was counted. The outcomes of PCI and CABG were obtained from the APPROACH database, and hospitalizations from the DAD.

### Statistical analysis

Descriptive statistics were used to describe study population characteristics. Subjects were considered to be “correctly” coded as obese if they had a diagnosis code of obesity in the administrative database and a BMI ≥ 30 kg/m^2^ in the measured data contained in APPROACH. The sensitivity, specificity, negative predictive value (NPV) and positive predictive value (PPV) of a diagnosis of obesity as coded in the administrative databases were determined using the physical measurement of obesity as the referent value. Each of these validity indices was calculated over the entire study time period as well as by year (2002 – 2008). The accuracy of coding was then considered across various demographic categories and clinical conditions.

As a second step, we assessed the relative impact of being coded as obese on outcomes, namely hospitalization, PCI or CABG, and mortality. We considered the association between obesity and outcomes in those correctly diagnosed as obese within the administrative data, those diagnosed as obese within APPROACH only, and those diagnosed as obese within APPROACH or the administrative database. The association between outcomes and obesity were evaluated in multivariate logistic regression models. The Odds Ratio (OR) between the outcomes and obesity and their 95% confidential intervals (95%CI) were estimated in logistic regression model while controlling for other factors. Statistical analysis was conducted using SAS Version 9.0.

The study protocol was approved by the ethics review board of the University of Calgary.

## Results

A total of 17,380 subjects were included in the initial analysis. Baseline characteristics of these patients are outlined in Table 1. The study population was largely male (56.4%) and 48.6% were aged ≥65 years old. Most subjects underwent coronary catheterization for urgent reasons, including myocardial infarction and unstable angina. In APPROACH, there were 3523 (20.3%) subjects with a BMI ≥ 30 kg/m^2^ (mean BMI of 32.84 kg/m^2^). Of these, 83.4% were obesity Class I, 13.5% were obesity Class II, and 3.1% were obesity Class III. In the DAD, 414 patients were coded as being obese.

**Table 1:** Baseline characteristics of the study population from clinical registry data (APPROACH)

	**Overall cohort**		**Obese by measured data**		**Obese by administrative data**	
	**Number of subjects (n)**	**Mean BMI (kg/m**^**2**^**) (±SD)**	**Number of subjects (n)**	**Mean BMI (kg/m**^**2**^**) (±SD)**	**Number of subjects (n)**	**Mean BMI (kg/m**^**2**^**) (±SD)**
**Total**	17380	29.96 ± 4.14	3523	32.84 ± 2.96	414	31.38 ± 4.57
**Age**						
<55	4341	27.24	950	32.93 ± 2.96	105	31.34 ± 4.30
55-65	4600	27.27	1009	32.79 ± 3.08	121	31.30 ± 4.93
65-75	4842	27.06	1023	32.75 ± 2.82	118	31.49 ± 4.80
> = 75	3597	26.11	541	32.94 ± 3.00	70	31.42 ± 4.00
**Gender**						
Female	5424	27.28	1535	33.72 ± 3.33	198	32.79 ± 4.88
Male	11956	26.82	1988	32.16 ± 2.43	216	30.10 ± 3.86
**Indication for coronary catheterization**						
Stable angina	4715	27.26	1015	32.75 ± 2.65	129	31.79 ± 3.35
Myocardial infarction	6883	26.86	1312	32.78 ± 3.05	163	31.32 ± 4.52
Unstable angina	3705	27.20	843	32.96 ± 3.08	91	30.50 ± 5.40
Other	2077	26.23	353	33.02 ± 3.15	31	32.64 ± 6.14
**Diabetes**						
Not diabetic	13717	26.70	2461	32.70 ± 2.86	243	30.99 ± 4.34
Diabetic	3663	27.94	1062	33.16 ± 3.15	171	31.95 ± 4.85
**Cerebrovascular disease**						
No history of CVD	16116	26.98	3258	32.84 ± 3.00	375	31.24 ± 4.53
History of CVD	1264	26.72	265	32.77 ± 2.48	39	32.75 ± 4.85
**Congestive heart failure**						
Absent			3062	32.78 ± 2.87	356	31.38 ± 4.40
Present			461	33.22 ± 3.49	58	31.41 ± 5.58
**Hypertension**						
Non-hypertensive	6215	26.38	974	32.64	119	31.48
Hypertensive	11165	27.29	2549	32.91	295	31.34
**Hyperlipidemia**						
Hyperlipidemic	4820	26.32	855	32.90 ± 2.90	93	31.85 ± 5.01
Non-hyperlipidemic	12560	27.21	2668	32.82 ± 2.98	321	31.25 ± 4.44
**Prior MI**						
No history of MI	13007	26.97	2664	32.84 ± 2.97	302	31.53 ± 4.40
Prior MI	4373	26.96	859	32.83 ± 2.92	112	31.00 ± 5.01

*Abbreviations:* SD Standard Deviation, *Obesity* BMI ≥30 kg/m^2^, *Obesity Class II* BMI ≥35 kg/m^2^, *Obesity Class III* BMI ≥ 40 kg/m^2^.

Rates of obesity remained stable year to year (see Table 2). The sensitivity of a diagnosis of obesity in the DAD was low at 7.75%. However, it was highly specific at 99.0%, with Negative Predictive Value (NPV) of 80.8% and a PPV of 65.9% (Table 2). There were minor variations in the sensitivity of an obesity diagnosis, under 10% throughout the study time period. There were no clear trends or improvements in sensitivity over time. Specificity and NPV were excellent throughout the study period, at over 98% and 80% respectively.

**Table 2:** Obesity prevalence and the validity of hospital discharge abstract (DAD) administrative health database coding of (n = 17380)

	**Prevalence in clinical registry, based on physical measure (n,%)**	**Prevalence in DAD, coded as obese (n,%)**	**Sensitivity**	**Specificity**	**NPV**	**PPV (%, 95% CI)**
Overall	3523 (20.27)	414 (2.38)	7.75	98.98	80.84	65.94 (61.38,70.51)
Year						
2002	607 (20.72)	85 (2.90)	8.24	98.49	80.41	58.82 (48.36, 69.29)
2003	517 (19.12)	80 (2.96)	9.48	98.58	82.16	61.25 (50.57, 71.93)
2004	564 (21.32)	67 (2.53)	8.69	99.14	80.02	73.13 (62.52, 83.75)
2005	499 (20.24)	42 (1.70)	5.81	99.34	80.6	69.05 (55.07, 83.03)
2006	466 (19.58)	39 (1.64)	6.44	99.53	81.38	76.92 (63.70, 90.15)
2007	443 (20.18)	41 (1.87)	5.87	99.14	80.64	63.41 (48.67, 78.16)
2008	427 (20.71)	60 (2.91)	9.37	98.78	80.67	66.67 (54.74, 78.59)
**Age**						
<55	950 (21.88)	105 (2.42)	7.58	99.03	79.27	68.57
55-65	1009 (21.93)	121 (2.63)	7.63	98.77	79.19	63.64
65-75	1023 (21.13)	118 (2.44)	8.02	99.06	80.08	69.49
> = 75	541 (15.04)	70 (1.95)	7.76	99.08	85.85	60.00
**Gender**						
Female	1535 (28.30)	198 (3.65)	9.84	98.79	73.52	76.26
Male	1988 (16.63)	216 (1.81)	6.14	99.06	84.11	56.48
**Indication for coronary catheterization**						
Stable angina	1015 (21.53)	129 (2.74)	8.77	98.92	79.81	68.99
Myocardial infarction	1312 (19.06)	163 (2.37)	7.85	98.92	82.01	63.19
Unstable angina	843 (22.75)	91 (2.46)	6.88	98.85	78.28	63.74
Other	353 (17.00)	31 (1.49)	6.52	99.54	83.87	74.19
**Diabetes**						
Not diabetic	2461 (17.94)	243 (1.77)	6.18	99.19	82.86	62.55
Diabetic	1062 (28.99)	171 (4.67)	11.39	98.08	73.05	70.76
**Cerebrovascular disease**						
No history of CVD	3258 (20.22)	375 (2.33)	7.49	98.98	80.85	65.07
History of CVD	265 (20.97)	39 (3.09)	10.94	99.00	80.73	74.36
**Congestive heart failure**						
Absent	3062 (20.35)	356 (2.37)	7.64	98.98	80.74	65.73
Present	461 (19.73)	58 (2.48)	8.46	98.99	81.48	67.24
**Hypertension**						
Non-hypertensive	974 (15.67)	119 (1.91)	7.80	99.18	85.27	63.87
Hypertensive	2549 (22.83)	295 (2.64)	7.73	98.86	78.36	66.78
**Hyperlipidemia**						
Hyperlipidemic	855 (17.74)	93 (1.93)	7.49	99.27	83.27	68.82
Non-hyperlipidemic	2668 (21.24)	321 (2.56)	7.83	98.87	79.91	65.11
**Prior MI**						
No history of MI	2664 (20.48)	302 (2.32)	7.43	98.99	80.59	65.66
Prior MI	859 (19.64)	112 (2.56)	8.73	98.95	81.60	66.96

*Abbreviations:NPV* Negative Predictive Value, *PPV* Positive Predictive Value, *CI* Confidence Interval.

Of those 414 subjects coded as obese in the DAD, nearly a third (141) were not actually obese when compared to measured data. These incorrectly coded subjects had a mean BMI of 26.9 kg/m^2^ (SD 3.6), were older (mean age 63.7 ± 10.8 years vs 62.7 ± 11.0, p-value = 0.3), less likely to be female (33.3% vs 55.3%, p < 0.0001), and more likely to be diabetic (35.5 vs 44.3%, p = 0.0827) than those who were correctly coded as obese in both administrative and clinical data (n = 273).

We further analyzed our data to assess whether or not demographic or clinical factors would influence the PPV of a DAD diagnosis of obesity (Table 2). The prevalence of obesity was higher in female subjects (28.3%) than male (16.6%), and the PPV was commensurately higher. The sensitivity of an administrative database obesity diagnosis was also higher in women. There were no trends across age groups, with the exception of a lower prevalence of obesity and PPV in the elderly (age >75 years) age group. The prevalence of obesity and the PPV was higher amongst those subjects with conditions associated with excess body mass: namely, diabetes and hypertension. This association was strongest for those with diabetes: the prevalence of obesity in patients with diabetes was 29.0%, versus 17.9% in those without; similarly, the PPV of an obesity diagnosis in the administrative database was higher amongst those with diabetes (70.8%) than those without (62.6%). The PPV did not seem to be impacted by a diagnosis of hyperlipidemia, congestive heart failure, or a history of previous myocardial infarction.

We further assessed whether BMI influenced the likelihood that a subject was coded as obese in the administrative database. Of those coded as obese within the administrative database, the large majority (72.9%) were Class I obese; of those not coded as obese, 84.3% were Class I obese, and 2.9% were Class III obese (Table 3). Thus, the higher the BMI by measured data the more likely subjects were to be coded as obesity in the administrative data.

**Table 3:** Obesity class amongst (1) those coded obese in the DAD administrative health database, and (2) those not coded obese in the DAD administrative health database

	**Obese in DAD**		**Coded as Obese in the DAD and by physical measure**		**Obese by physical measure and not coded obese in administrative data**		**Obese by physical measure**	
	**Number**	**Percent**	**Number**	**Percent**	**Number**	**Percent**	**Number**	**Percent**
Total	414	100	273	100	3250	100	3523	100
Weight Class								
BMI <30	141	34.06	0	0	0	0	0	0
I (BMI 30-34.99 kg/m^2^)	199	48.07	199	72.89	2740	84.31	2939	83.42
II (BMI 35-39.99 kg/m^2^)	60	14.49	60	21.98	416	12.8	476	13.51
III (BMI ≥ 40 kg/m^2^)	14	3.38	14	5.13	94	2.89	108	3.07

*Abbreviations:BMI* Body Mass Index.

As a final step, we wished to determine if obesity as coded in DAD was differentially associated with outcomes in comparison to obesity as determined by physical measurement. Over the course of the study, there were 7547 hospital admissions, 10772 CABG and PCIs, and 703 deaths. In subjects who were obese by DAD, there was no significant association between obesity and re-hospitalization or mortality (Table 4). The same was demonstrated for those “correctly” coded as obese. Considering those subjects who were obese by physical measurement only, obesity was associated with decreased risk of mortality or re-hospitalization, but an increased risk of repeat procedure. The same was seen for subjects who were obese by physical measurement or administrative data.

**Table 4:** The impact of obesity on one-year outcomes

**Comparison groups**	Odds ratio (95%CI)*		
	**Re-hospitalization**	**CABG or PCI**	**Mortality**
Obese by DAD data only (non-obese n = 16966, obese n = 414)	0.84 (0.68, 1.03)	2.26 (1.77, 2.88)	0.74 (0.42, 1.32)
Obese by physical measurement and DAD (non obese = 17107, obese n = 273)	0.90 (0.70, 1.15)	2.81 (2.07, 3.81)	0.60 (0.28, 1.30)
Obese by physical measurement only (non-obese n = 14130, obese 3250)	0.84 (0.78, 0.91)	1.20 (1.10, 1.31)	0.62 (0.49, 0.78)
Obese by physical measurement or DAD (non-obese n = 13857, obese 3523)	0.83 (0.77, 0.90)	1.22 (1.12, 1.32)	0.63 (0.51, 0.79)

Obesity classified by data source: hospital discharge abstract (DAD) administrative health database and clinical registry physical measurement (N = 17380).

*Adjusted for sex, age group (by decade), and co-morbidity (0, 1-4, or ≥ 5 of chronic obstructive pulmonary disease, cerebrovascular disease, renal disease, congestive heart failure, dialysis, hypertension, hyperlipidemia, previous myocardial infarction, peripheral vascular disease, and diabetes).

## Discussion

We have confirmed the findings of previous investigators that administrative data under-coded obesity as a diagnosis. However, once obesity is coded in the data, it is coded relatively accurately, as for other chronic conditions [10]. Administratively captured obesity was more likely in patients with higher classes of obesity or obesity-related complications. Interestingly, despite our suspicion that obesity coding would improve over time with increasing general awareness of the relationship between obesity and disease we found no evidence for this. These finding suggest that administrative databases could not be used for obesity surveillance due to under-reporting but could potentially be used to identify obesity for forming a cohort of obese subjects for follow-up studies.

Despite the general poor capture of obesity in administrative databases, we did find a number of conditions under which obesity is better captured. The PPV of an obesity diagnosis is higher in women than in men, and it is also higher in a number of conditions that are known to be associated with obesity, namely diabetes and hypertension. Additionally, in those cases in which obesity is actually captured, it is captured with great accuracy, as demonstrated by the high PPV seen in this study. However the obese subjects as captured by the administrative database are more likely to be Class III obese than those who are not captured, i.e. there is a bias towards coding those with a higher BMI as obese, missing those who are Class I obese. Thus, the administrative databases are capturing subjects who perhaps already have complications from their obesity, as evidenced by the fact that the PPV of an obesity diagnosis in the administrative database is higher in those with obesity related complications such as diabetes. Cohorts defined using administrative data may therefore show a falsely high correlation between obesity and the development of complications or poor outcomes, as the obese subjects correctly identified in administrative databases are potentially sicker than an average obese subject.

Some work has been done assessing the validity of obesity coding in administrative data in previous studies. In one chart based study by Quan et al, only weight loss, coagulopathy and blood loss anemia were less validly coded than obesity in administrative data. In Switzerland, obesity was under-coded (prevalence 2.2% in 1999, 3.2% in 2001 and 4.1% in 2003) compared with the prevalence in chart ranging 6.6-7.3% but coding improved over years (sensitivity 29.4% in 1999, 39.5% in 2001 and 51.5% in 2003; PPV 92.%, 81.1% and 91.7% in these years, respectively) [13]. Reasons put forth for the poor capture of obesity in administrative data include the fact that obesity is not explicitly mentioned in either physician or nursing notes, and also that coders may intentionally not code diagnoses such as obesity owing to time constraints when doing data abstraction. In the limited time for coding each chart, coders are likely to ignore risk factors, focusing on overt clinical conditions. Coding guidelines pay more attention to conditions contributing to resource use and the use of extra resources by obese subjects is a topic that is only more recently understood [14]. In addition, physicians may not explicitly mention obesity in the chart summary page which coders mainly rely on, as obesity is poorly recognized as a disease. BMI was also not well-documented although height and weight are available on most clinical charts. The diagnosis of obesity is often made based on clinician’s subjective observation, likely capturing higher class obesity. If administrative database data abstractors are coding height and weight in the chart, rates of obesity are likely to be accurate.

Another difficulty in defining obesity is the use of patient self-reported data. On patient admission to hospital, height and weight are frequently determined by patient report, and this information is then recorded in the patient record. It has been shown that patients overestimate their height and underestimate their weight, which leads to underestimates of the prevalence of obesity. This misrepresentation of BMI is more common in the obese [15].

A recent study by Woo et al [16] considered both hospital administrative data and a clinical database that captured height and weight for all children admitted to hospital. The administrative database failed to capture obesity for the majority of obese children who were admitted to hospital. A diagnosis of obesity in the administrative database only had an 8% sensitivity based on their BMI. More importantly, when outcomes were compared between non-obese children and obese children based on (a) obesity as captured in the administrative database versus (b) obesity as captured in the clinical database, the impact of obesity was found to be different. A diagnosis of obesity recorded in the administrative data identified “sporadic, potentially non-representative, hospital discharges with shorter lengths of stay.” However our study demonstrated that the association between obesity and each of the outcomes (hospitalization, PCI/CABG or death) were similar between regardless of how obesity was coded. Differences arose in terms of the significance (for mortality, likely due to the small number of deaths, and for re-hospitalization), and in terms of the magnitude for PCI/CABG.

For missing information on obesity in administrative data, merging with clinical databases such as was done in this study is an important way by which to enhance the quality data found in administrative databases. Additionally, physician claims databases as well as prescription databases are potential sources of obesity information. In a review of available literature, nearly all studies of obesity using larger databases are not based on administrative databases alone. This includes papers from NHANES assessing obesity prevalence [17,18], studies assessing the association between adiposity and cardiovascular outcomes, [19-25] and studies assessing care in obese subjects [26,27]. For instance, in a paper by Chang et al., while Medicare claims and enrollment were used to assess for service utilization, data on BMI were obtained from a merge with the Medicare Current Beneficiary Survey (MCBS). Similar studies done strictly using administrative or claims data without data enrichment to determine BMI would only identify a high risk group of obese subjects [28].

### Limitations

There are a number of limitations in this study that need be noted. Firstly, we have only considered cardiac patients. As cardiovascular disease is a complication related to obesity, rates of obesity coding in administrative data may be higher among this population than in the general inpatient population. However, in a study by Quan et al assessing a random sample of charts, obesity had prevalence 2.7% in ICD-9-CM DAD and 1.9% in ICD-10 DAD [10] similar to the rate seen in this population. We could also only consider the impact of coding on outcomes in cardiac populations; more distinct patient populations need to be assessed.

## Conclusions

A call to arms has been put forth by organizations such as the American Heart Association, recognizing that health care providers have not done a good job assessing for obesity and suggesting “the measurement and documentation of BMI in all adults” [29,30]. In this study we have demonstrated that even when obesity is present, care givers and coders do a poor job documenting its presence – though subjects at highest risk of complications are accurately identified. This large study demonstrates three key pieces of information: obesity is underreported in administrative data with low sensitivity, and hence cannot be used for incidence and prevalence surveillance; obesity coding in administrative databases could be used to define a cohort for follow-up or outcomes studies, supported by high PPV and similar outcomes conclusions between two databases; finally, we strongly recommend adding height and weight into routine administrative data coding, as is done age and sex. This would make these data an invaluable resource for studies of obesity and population health.

## Abbreviations

APPROACH: Alberta Provincial Project for Outcomes Assessment in Coronary Heart Disease; BMI: Body mass index; CABG: Coronary artery bypass grafting; CVD: Cerebrovascular disease; DAD: Discharge abstract database; ICD-9-CM: International classification of diseases, ninth revision, clinical modification; MI: Myocardial infarction; NPV: Negative predictive value; PCI: Percutaneous coronary intervention; PPV: Positive predictive value.

## Competing interests

The author(s) declare that they have no competing interests.

## Authors’ contributions

BJM conceived the project, gathered and concatenated databases, wrote the manuscript. GC conducted data analysis and assisted in manuscript revisions. MG assisted in data interpretation and manuscript revisions. HQ assisted in conceiving the project and edited the manuscript. All authors read and approved the final manuscript.

## Authors’ information

BJM is a cardiac surgery resident at the University of Calgary. GC is an associate professor in community health sciences at the University of Calgary. MG is a clinical cardiologist, clinical professor and member of the APPROACH research group at the University of Alberta. HQ Alberta Heritage Foundation for Medical Research Population Health Investigator and an Associate Professor at the Department of Community Health Sciences and the Centre for Health and Policy Study of the University of Calgary.

## Pre-publication history

The pre-publication history for this paper can be accessed here:

http://www.biomedcentral.com/1472-6963/14/70/prepub
